# Heart Rate Dependency and Inter-Lead Variability of the T Peak – T End Intervals

**DOI:** 10.3389/fphys.2020.595815

**Published:** 2020-12-15

**Authors:** Irena Andršová, Katerina Hnatkova, Martina Šišáková, Ondřej Toman, Peter Smetana, Katharina M. Huster, Petra Barthel, Tomáš Novotný, Georg Schmidt, Marek Malik

**Affiliations:** ^1^Department of Internal Medicine and Cardiology, University Hospital Brno, Faculty of Medicine, Masaryk University, Brno, Czechia; ^2^National Heart and Lung Institute, Imperial College London, London, United Kingdom; ^3^Wilhelminenspital der Stadt Wien, Vienna, Austria; ^4^Klinikum rechts der Isar, Technische Universität München, Munich, Germany

**Keywords:** T wave peak, T wave spatial loop, heart rate dependency, ECG lead comparison, sex differences

## Abstract

The electrocardiographic (ECG) assessment of the T peak–T end (Tpe) intervals has been used in many clinical studies, but several related physiological aspects have not been reported. Specifically, the sources of the Tpe differences between different ECG leads have not been systematically researched, the relationship of Tpe duration to underlying heart rate has not been firmly established, and little is known about the mutual correspondence of Tpe intervals measured in different ECG leads. This study evaluated 796,620 10-s 12-lead ECGs obtained from long-term Holters recorded in 639 healthy subjects (311 female) aged 33.8 ± 9.4 years. For each ECG, transformation to orthogonal XYZ lead was used to measure Tpe in the orthogonal vector magnitude (used as a reference for lead-to-lead comparisons) and to construct a three-dimensional T wave loop. The loop roundness was expressed by a ratio between its circumference and length. These ratios were significantly related to the standard deviation of Tpe durations in different ECG leads. At the underlying heart rate of 60 beats per minute, Tpe intervals were shorter in female than in male individuals (82.5 ± 5.6 vs 90.0 ± 6.5 ms, *p* < 0.0001). When studying linear slopes between Tpe intervals measured in different leads and the underlying heart rate, we found only minimal heart rate dependency, which was not systematic across the ECG leads and/or across the population. For any ECG lead, positive Tpe/RR slope was found in some subjects (e.g., 79 and 25% of subjects for V2 and V4 measurements, respectively) and a negative Tpe/RR slope in other subjects (e.g., 40 and 65% for V6 and V5, respectively). The steepest positive and negative Tpe/RR slopes were found for measurements in lead V2 and V4, respectively. In all leads, the Tpe/RR slope values were close to zero, indicating, on average, Tpe changes well below 2 ms for RR interval changes of 100 ms. On average, longest Tpe intervals were measured in lead V2, the shortest in lead III. The study concludes that the Tpe intervals measured in different leads cannot be combined. Irrespective of the measured ECG lead, the Tpe interval is not systematically heart rate dependent, and no heart rate correction should be used in clinical Tpe investigations.

## Introduction

Detailed classification and quantification of repolarization abnormalities is one of the unmet needs of contemporary electrocardiography. While noticeable progress has been made in the understanding of the electrocardiogram (ECG) manifestations of congenital channelopathies (Zareba, 2006) and of drug-induced ion channel abnormalities (Fenichel et al., 2004) as well as in the methodology of QT interval measurement and of its heart rate correction (Garnett et al., 2012), comprehension of the details of T wave changes due to ischemic heart disease and nonischemic cardiomyopathies remains elusive. The terms of “nonspecific T wave changes” is frequently used to describe ECGs in which the T wave does not appear normal but for which the present knowledge does not allow the details of the underlying electrophysiological abnormality to be identified.

Since both spatial and temporal repolarization abnormalities are linked to arrhythmogenesis, different methods have previously been proposed to quantify repolarization heterogeneity based on simple measurements applicable to standard 12-lead ECG recordings. Some three decades ago, the concept of the so-called QT dispersion (that is the lead-to-lead variability of QT interval duration) became very popular (Day et al., 1990; Okin et al., 2000) only to be quickly recognized as a mere expression of measurement inaccuracies and errors more frequent with abnormal rather that normal recordings (Kors and van Herpen, 1998; Kors et al., 1999; Rautaharju, 1999; Malik and Batchvarov, 2000) but without any physiological link to repolarization heterogeneity (Malik et al., 2000; Smetana et al., 2011).

More recently, previous observations made with canine wedge preparations (Sicouri and Antzelevitch, 1991; Antzelevitch, 2008) have been interpreted as a suggestion that the interval between the peak and the end of the T wave (Tpe interval) can serve as a measure of repolarization heterogeneity. A recent, albeit somewhat limited meta-analysis concluded that the Tpe interval is “a useful risk stratification tool in different diseases and in the general population” (Tse et al., 2017). Nevertheless, a closer inspection of the different studies published on the usefulness of the Tpe interval shows substantial inconsistencies in the measurement (e.g., different ECG leads and/or different combinations of measurements across several leads) as well as in the use of both heart rate uncorrected and heart rate corrected Tpe intervals (Malik et al., 2018). There is no consensus on whether the measurements in different ECG leads are mutually equivalent. In addition, systematic data are lacking on the heart rate dependency of Tpe intervals measured in different leads.

To address these questions, we have analyzed Tpe measurements in different ECG leads across a large collection of long-term 12-lead ECG recordings in healthy subjects. As these recordings included episodes of different heart rates, we were also able to study not only the heart rate dependency of the Tpe intervals but also the heart rate dependency of lead-to-lead differences.

## Materials and Methods

### Investigated Population and Electrocardiographic Recordings

A collection of Holter recordings previously analyzed for a different purpose was used (Toman et al., 2020). Altogether, 639 healthy adult subjects participated at six clinical pharmacology studies. All subjects had a normal resting ECG and normal clinical investigation before enrollment as mandated in clinical pharmacology research (Guideline, 2001). Standard inclusion and exclusion criteria applicable to Phase I pharmacology studies applied (Guideline, 2001). Among others, negative tests of recreational substances and negative pregnancy tests for female subjects were required. All the source studies were ethically approved by the institutional ethics bodies (Focus in Neuss; Parexel in Baltimore, Bloemfontein, and Glendale; PPD in Austin; and Spaulding in Milwaukee). All subjects gave informed written consent to the participation according to the Helsinki declaration.

As previously described (Toman et al., 2020), each of the studies included repeated 12-lead day-time Holter recordings. In each participant, the recordings were made during multiple baseline days when the subjects were off any medication, did not smoke, and refrained from consuming caffeinated drinks. During these baseline days, study protocols included repeated positioning maneuvers aiming at capturing wide heart rates ranges in each participant. The Holter recordings used Mason–Likar electrode positions. The right arm (RA) and left arm (LA) electrodes were placed on top of or close to the acromioclavicular joints; the left foot (LF) and neutral electrodes were placed on top of or close to the iliac crests.

Clinical conduct of the baseline days did not include any aspects that would make the data incompatible or incomparable between the source studies. As the investigation described in this text utilized only drug-free baseline recordings, details of the clinical pharmacology investigations are not relevant.

### Electrocardiographic JT Interval Measurements

Using previously developed technology combining computerized signal processing with visual checks and manual corrections of the measurements (Malik et al., 2008a, 2012; Toman et al., 2020), multiple 10-s segments were extracted from each of the Holter recordings aiming at the inclusion of segments with different underlying heart rates. For each extracted segment, a 5-min history of preceding RR intervals was obtained.

Each extracted 10-s ECG segment was filtered to reduce noise pollution and to eliminate baseline wander (Malik et al., 2008a, 2012). Subsequently, a representative median beat was constructed (Xue, 2009), sampled at 1,000 Hz. In this representative beat, all 12 leads were superimposed on the same isoelectric axis, and QRS offset and T offset points were identified using algorithms that were previously developed and described (Malik et al., 2008a, 2012). The quality control of the measurement of these points included visual verification and manual correction of computerized ECG processing by at least two independently working cardiologists with subsequent independent reconciliation in case of measurement disagreement. Pattern matching algorithms (Hnatkova et al., 2009) were also applied. This ensured that comparable morphologies of QRS offset and T offset were measured systematically. The visual verification and manual correction of the T offset measurements also distinguished between T and U waves. The shallow U waves frequently seen in precordial leads of normal ECGs were excluded from subsequent T wave analyses.

### T Wave Loop Construction

Using the previously published conversion matrix suitable for the Mason–Likar electrode positions (Guldenring et al., 2012), the voltage values of the representative beats of each ECG sample were used to derive orthogonal XYZ leads. From these, vector magnitude lead *VM* was constructed using the standard formula of $VM\left(t\right)={\left(X_{t}^{2}+Y_{t}^{2}+Z_{t}^{2}\right)}^{1/2}$, where *W*_*t*_ is the voltage of the orthogonal lead *W* at the time instant *t*.

The orthogonal XYZ leads were also used to construct the T wave loop as a three-dimensional curvature starting and ending at the (0, 0, 0) point and passing sequentially through the points [*X*(*t*), *Y*(*t*), *Z*(*t*)] for *t* ranging from *J* (the QRS offset) to *T* (the T wave offset). The length of the T wave loop was calculated as a simple sample-to-sample sum of the distances between neighboring points $ℒ=VM\left(J\right)+\sum_{t=J}^{T-1}{\left[{\left(X_{t+1}-X_{t}\right)}^{2}+{\left(Y_{t+1}-Y_{t}\right)}^{2}+{\left(Z_{t+1}-Z_{t}\right)}^{2}\right]}^{1/2}+VM\left(T\right)$. [Note that the formula ensures that the loop starts and end at the (0, 0, 0) point.] To express the roundness of the loop, the T loop ratio was calculated as $ℒ/\left[2\times \max_{J\leq t\leq T}VM\left(t\right)\right]$. This ratio is 1 for T wave loops that are strictly unidimensional and collapsed into a line, while an increasing value of the ratio signifies loops of increasing roundness (or of even more complex morphology).

### T Peak Measurements

In each ECG lead, in the orthogonal vector magnitude as well as in the derived nonstandard ECG dipoles (see the details described further), the same previously published algorithm (Johannesen et al., 2016) was used to detect the peak of the T wave within the interval between the QRS offset and T wave offset.

Objective noise assessment algorithms (Batchvarov et al., 2002) were used to eliminate ECG leads in which the T wave morphology was too noise polluted to allow the T peak detection with sufficient confidence. To exclude leads with flat T waves in which the result of T peak detection algorithms might have been questioned, measurements were accepted only in those leads in which the voltage of the detected T peak differed from the line connecting the QRS offset and T wave offset by at least 100 μV and in which the T peak detection was stable. When dual peaks of opposite orientation were detected in biphasic T waves, the peak with the highest absolute voltage was used. Nevertheless, biphasic T waves were seen almost exclusively only in the derived precordial bipoles (as explained further in this text).

In each lead, the Tpe interval was measured as the difference between the T peak and the T wave offset (common to all leads of the same ECG sample).

### Underlying Heart Rate

To study the relationship of the Tpe durations to the underlying heart rate, hysteresis-corrected RR interval values were used. Based on existing experience, previously published exponential decay hysteresis model (Malik et al., 2008b) was used based on the following considerations: For a Tpe interval measurement, the sequence of preceding RR intervals ${\left\{RR_{i}\right\}}_{i=0}^{N}$ (*RR*_0_ closest to the Tpe measurement) is considered. The RR interval representing the heart rate underlying the Tpe measurement is then calculated as

$$RR^{\prime }=\sum_{i=0}^{N}\omega_{i}RR_{i}$$

where for each *j* = 0, …, *N*,

$$\sum_{i=0}^{j}\omega_{i}=\frac{\left(1-e^{-\lambda \frac{\sum_{i=0}^{j}RR_{i}}{\sum_{i=0}^{N}RR_{i}}}\right)}{\left(1-e^{-\lambda }\right)}.$$

The coefficient λ characterizes the profile of the Tpe/RR hysteresis, i.e., the speed with which Tpe interval adapts to changing heart rate. While subject-specific optimization of the coefficient λ is possible, the lead-to-lead comparison would become problematic if such a subject-specific optimization was performed since it would need to be applied to different ECG leads separately. We have therefore used a common value of λ = 7.4622, which corresponds to the 95% adaptation after a 2-min period (Malik et al., 2016). Note also that the majority of the extracted ECG segments were preceded by a stable heart rate; when all preceding RR intervals are of practically the same duration, the hysteresis-corrected RR′ value corresponds to the common RR duration.

### Data Investigations

To analyze the relationship of the Tpe intervals measured in different ECG leads and to investigate their heart rate relationship, we utilized the available data in three separate facets of the study.

#### Spread of the Lead-to-Lead Tpe Values

The principles of electrocardiography suggest that any lead-to-lead differences are caused, especially in normal physiologic situations, by different vector projections of the same spatial distribution of electrophysiological processes rather than by associations of different ECG leads with different myocardial regions. When applied to the lead-to-lead differences of the Tpe intervals, this principle suggests that Tpe spread across leads increases with the spatial spread of the T wave loop.

To test this suggestion, we investigated the relationship between the standard deviation (SD) of Tpe durations in different leads and the T loop ratio. Specifically, for each study subject, the mean value of the T loop ratio of all extracted ECG segments was related to the mean of SD of Tpe durations in different ECG leads. This use of subject-specific average avoided the problem of influencing the relationships by multiple data from the same subject that could not be considered as mutually independent.

**FIGURE 1:**
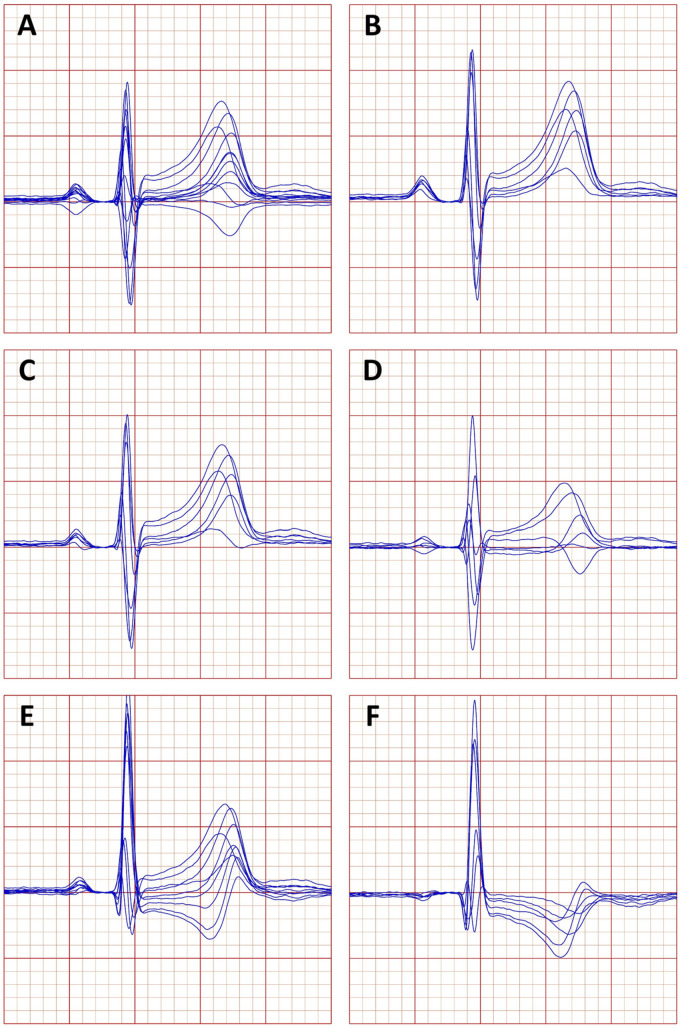
Example of a representative beatform of an electrocardiogram (ECG) obtained in a 33-year-old male. **(A)** Standard leads of the 12 lead ECG, **(B)** the bipolar leads between the precordial electrodes and the right arm electrode, **(C)** the bipolar leads between the precordial electrodes and the left arm electrode, **(D)** the bipolar leads between the precordial electrodes and the left foot electrode, **(E)** the “wide” precordial dipoles, and **(F)** the “narrow” precordial electrodes (see the text for explanation of the precordial dipoles).

For the purpose of this investigation, six different sets of ECG leads were considered (Figure 1):

• All the 12 standard leads of the ECG,
• Six bipolar leads between the V1 to V6 electrodes and the RA electrode,
• Six bipolar leads between the V1 to V6 electrodes and the LA electrode,
• Six bipolar leads between the V1 to V6 electrodes and the LF electrode,
• Bipolar leads V1–V2, V1–V3, …, V1–V6, and V–V3, V2–V4, …, V2–V6, forming a group of nine “wide” precordial dipoles, and
• Bipolar leads V3–V4, V3–V5, V3–V6, V4–V5, V5–V6, and V5–V6, forming a group of six “narrow” precordial dipoles.

The nonstandard bipolar leads were derived from the standard 12 leads using trivial algebraic equations.

#### Heart Rate Dependency of the Tpe Values

Because of the known subject-specific relationship between the QT intervals and the underlying heart rate, the heart rate dependency of the Tpe intervals measured in different leads was investigated in each study subject separately. That is, for each subject, linear regressions between Tpe intervals measured in different standard ECG leads and the underlying heart rate were calculated. The slopes of these intrasubject regressions were statistically summarized for each standard ECG lead.

The intrasubject linear regressions also allowed to project the Tpe intervals in the given subject to heart rates of 60 and 120 beats per minute (bpm). These projections were repeated for the different leads and statistically summarized to express the influence of the heart rate changes on the Tpe durations.

The residuals of the linear regressions also allowed us to study intrasubject reproducibility of Tpe interval measurements. Since the residuals are influenced by the magnitude of the dependent variable, we used the relative residuals that we defined, in each study subject, as the proportion between the Tpe/RR residual and the projected value of Tpe at the heart rate of 60 bpm.

The same study of linear regressions was also repeated for the Tpe interval measured in the orthogonal XYZ vector magnitude and for the interval between the QRS offset and T wave offset (the JT interval), which is lead independent.

To investigate whether the heart rate influence on the Tpe intervals measured in different leads is physiologically driven by similar processes as those underlying the heart rate influence on the JT interval, the 60–120-bpm changes in the Tpe intervals were related to the 60–120-bpm changes in the JT intervals in the corresponding subjects.

#### Reconstruction of Orthogonal Vector Magnitude Tpe From Standard ECG Leads

Consistent with previous studies (Johannesen et al., 2014; Hnatkova et al., 2019a), it seems reasonable to propose that the peak of the T wave detected on the vector magnitude of orthogonal XYZ leads represents the instance of maximum repolarization changes across ventricular myocardium. This point might therefore be possibly proposed for the gold standard expression of the Tpe interval.

Consequently, we have investigated whether the Tpe interval measured in the vector magnitude of orthogonal XYZ leads can be reasonably approximated by an algebraic combination of Tpe intervals measured in standard ECG leads. For this purpose, we selected those standard ECG leads in which the T peak was measurable in a majority of the analyzed ECG segments. Multivariable regression analysis was subsequently performed to calculate linear regression coefficients that would allow to estimate the Tpe interval of the XYZ vector magnitude from the measurements in standard leads. Two calculations of this multivariable regression analysis were performed that did and did not include underlying heart rates and a constant intercept value.

The multivariable regression coefficients were obtained based on the analysis of all ECG samples in which the Tpe interval was measurable in all selected standard ECG leads. Subsequently, the actual precision of the Tpe of XYZ vector magnitude reconstruction was assessed in each study subject separately by obtaining the mean and the SD of the differences between actual measurements and the corresponding reconstructed values.

### Sex Differences

Because of the known sex differences between QT intervals and QT/RR relationships, all the statistical summaries of the study were preformed separately for the subgroups of female and male participants.

### Statistics and Data Presentation

Data are presented as means ± SD. Differences between female and male subjects were evaluated using two-sample, two-tail *t*-test assuming different variances of compared samples. Intrasubject comparisons (e.g., comparisons between Tpe intervals projected to heart rates of 60 and 120 bpm) were evaluated using two-tail paired *t*-test. *p*-Values above 0.05 were considered statistically nonsignificant (NS). Because of the interdependency of evaluated data, no correction for multiplicity of testing was performed, and all statistical tests performed are presented. Statistical evaluation used the IBM SPSS package version 25.

## Results

### Population and Electrocardiographic Measurements

The source clinical pharmacology studies investigated 639 subjects (311 female). The ages of sex-defined subgroups were practically identical (female, 33.8 ± 10.1 years; male, 33.9 ± 8.7 years, NS).

The study involved measurement of 796,620 ECG samples of which 385,135 and 411,485 were obtained in female and male subjects, respectively. The subject-specific counts of ECG samples were practically the same in female and male subjects (1,238 ± 253 vs 1,262 ± 240, respectively).

Both the intrasubject maximum and minimum heart rates (hysteresis corrected) of the ECG segments in female subjects (115.0 ± 13.0 and 53.7 ± 6.3 bpm, respectively) were significantly faster than those in male subjects (109.0 ± 13.1 and 50.3 ± 5.5 bpm, respectively, both *p* < 0.0001 for comparison with female subjects). The intrasubject ranges between minimum and maximum heart rates were also wider in female compared to male subjects (61.3 ± 12.6 vs 58.7 ± 12.6, *p* = 0.009). Nevertheless, these intrasubject ranges were sufficiently wide so that regression projections to heart rates of 60 and 120 bpm involved, where necessary, stable extrapolations.

**FIGURE 2:**
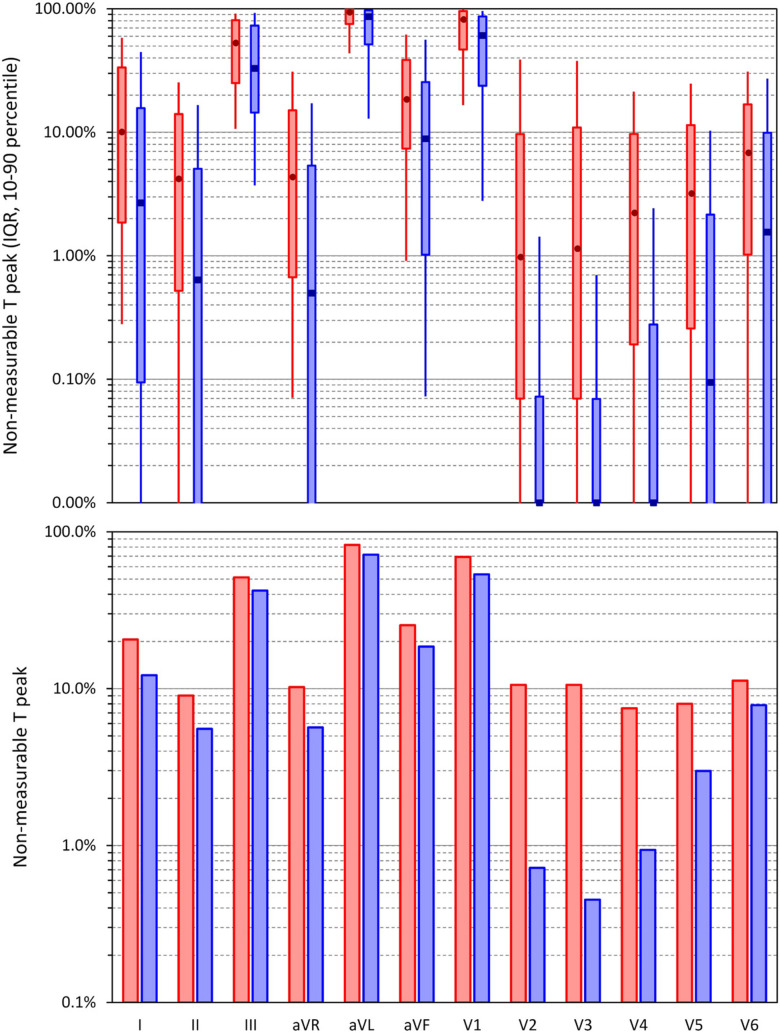
Incidence of non-measurable peaks of the T wave in standard electrocardiogram (ECG) leads. The bottom panel shows the incidence among all ECG segments investigated in the study pooled together; the top panel shows the summary of the incidence in individual study subjects—the bars show the interquartile ranges, and the error bars the spreads between the 10th and 90th percentiles of the population. The dark marks in the middle of the bars are the population medians. In both panels, the displays in red and blue show the data in female and male subjects, respectively. Note the logarithmic vertical axes (in the top panel, there were only 0 display values below 0.01%).

While the Tpe interval of XYZ vector magnitude was measurable in all selected segments (since the selection excluded noise polluted segments), Figure 2 shows that the measurability of the T peak differed substantially in different leads. Failed localization of T peak due to flat and/or too widely spread T waves was frequent in leads III, aVL, and V1 in which the T peak was measurable only in 48.8, 17.4, and 30.8% of ECG segments in female subjects and in 57.8, 28.5, and 46.4% of ECG segments in male subjects, respectively. (While it was possible to identify T wave peaks in these leads, the number of the accepted measurements was reduced by failed consistency and repeatability checks.) In leads II, aVR, V2, V3, V4, V5, and V6, T peak was measurable in more than 90% of all ECG segments, and as seen in Figure 2, T peak was more frequently measurable in male compared to female subjects. As also seen in Figure 2, the proportions of T peak measurability observed in the complete data were replicated also in the data of individual study subjects.

### Spread of the Lead-to-Lead Tpe Values

**FIGURE 3:**
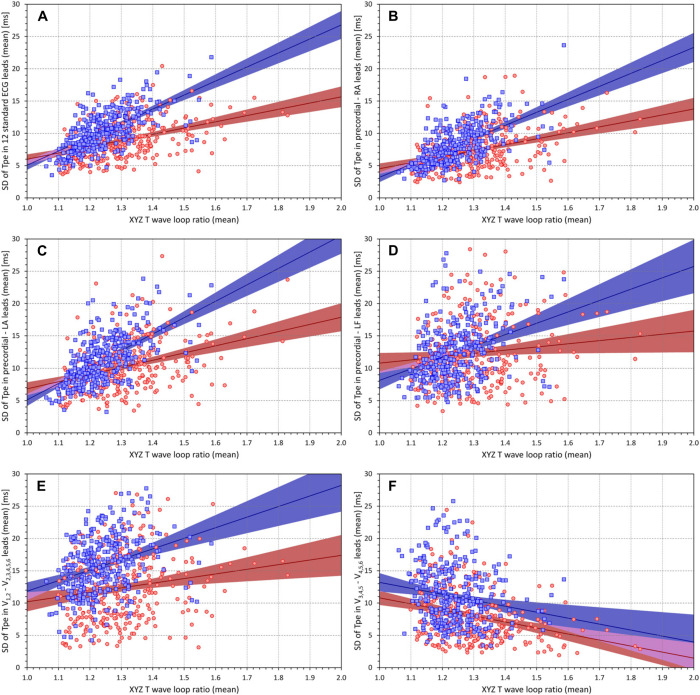
Scatter diagrams between the T wave loop ratios (mean values in individual subjects) and the standard deviations of the Tpe intervals in groups of electrocardiogram (ECG) leads (mean values in individual subjects). Different panels of the figure correspond to different groups of ECG leads–the association of the panels with the lead groups is the same as in Figure 1. In each panel [please see the labels of vertical axes for the explanation of panels **(A–F)**], the red circles and blue squares correspond to female and male subjects, respectively. The solid red and solid blue lines show the linear regressions between the measured standard deviations of Tpe intervals and the T wave loop ratios in female and male subjects, respectively. The red- and blue-shaded areas are the 95% confidence intervals of the regression lines; the violet areas are the overlaps between the confidence intervals of the sex-specific regressions. SD, standard deviation; RA, right arm; LA, left arm; LF, left foot.

**FIGURE 4:**
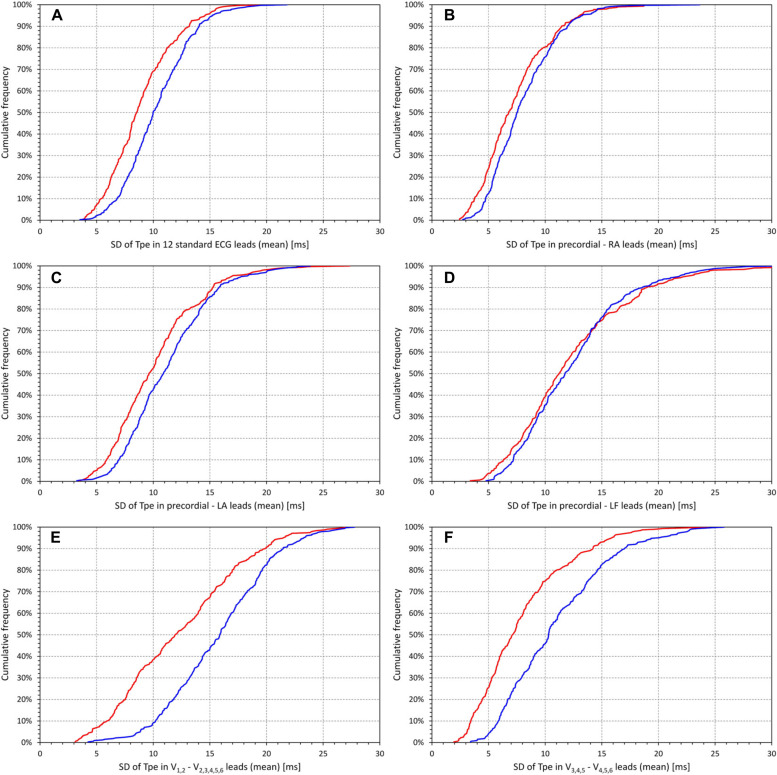
Cumulative distributions of the standard deviations of the Tpe intervals in groups of electrocardiogram (ECG) leads (mean values in individual subjects). Different panels of the figure correspond to different groups of ECG leads–the association of the panels with the lead groups is the same as in Figure 1. In each panel [please see the labels of horizontal axes for the explanation of panels **(A–F)**], the red and blue lines correspond to the distributions in female and male subjects, respectively. SD, standard deviation; RA, right arm; LA, left arm; LF, left foot.

Figure 3 shows the scatter diagrams between the intrasubject means of T wave loop ratio and the corresponding intrasubject means of the ECG-specific SDs of the Tpe intervals in the standard 12 leads (Figure 3A), in bipolar leads between precordial electrodes and the RA, LA, and LF electrodes (Figures 3B–D, respectively), in the wide precordial bipolar leads (Figure 3E), and in the narrow precordial bipolar leads (Figure 3F). Corresponding cumulative frequencies of the intrasubject means of the ECG-specific SDs of the Tpe intervals are shown in Figure 4.

As seen in Figure 3, all sets of the ECG leads, except for the narrow precordial bipoles, showed statistically significant positive relationship with the T wave loop ratio. The result for the set of narrow precordial bipolar leads was the opposite with statistically significant negative relationship. This might possibly be surprising but is likely caused by restricted projections of the T wave loop combined with isoelectric projections of narrow loops.

Figure 3 also shows that the relationship to the T wave loop ratio was steeper in male compared to female subjects (again with the exception of narrow precordial bipoles). Figure 4 shows that the interlead spread of Tpe intervals (i.e., of the T peak positions) among the standard ECG leads as well as among the precordial bipoles was more compact in female compared to male subjects. While the SDs of Tpe intervals in the precordial–LF dipoles were similar between female and male subjects, in other lead groups, the spread of the Tpe intervals was larger in male compared to female subjects.

### Heart Rate Dependency of the Tpe Values

**FIGURE 5:**
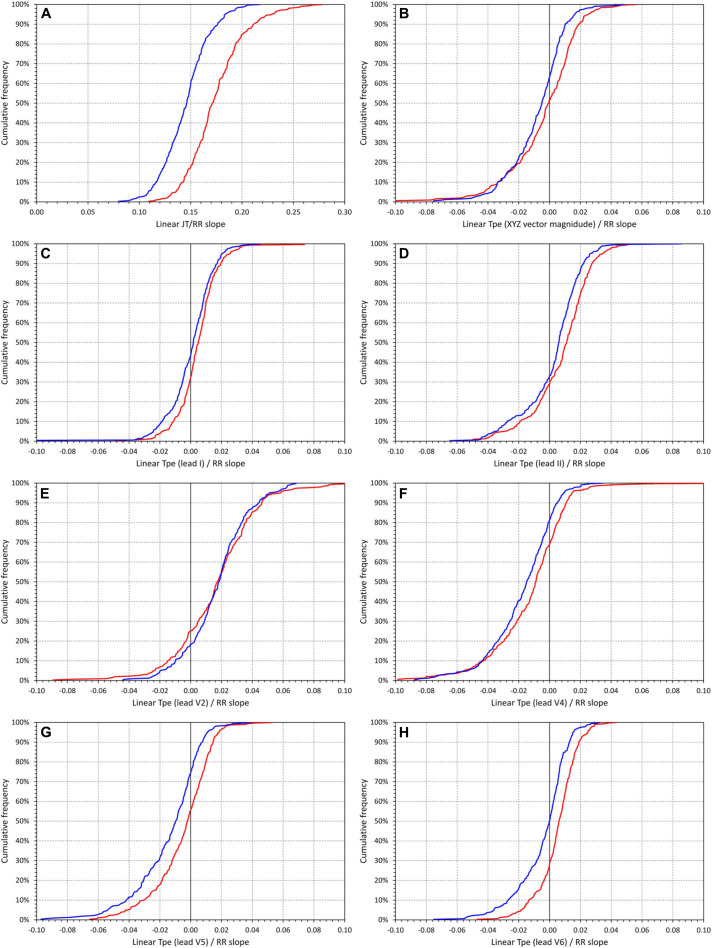
Cumulative distributions of the intrasubject slopes of linear regressions between underlying hysteresis corrected heart rate and JT intervals **(A)** and Tpe intervals measured in different electrocardiogram (ECG) leads [**(B)** orthogonal XYZ vector magnitude, **(C)** lead I, **(D)** lead II, **(E)** lead V2, **(F)** lead V4, **(G)** lead V5, and **(H)** lead V6]. In each panel, the red and blue lines correspond to the distributions in female and male subjects, respectively. Note that while in **(A)**, the horizontal axis ranges between 0 and 0.30, the horizontal axes in all other panels range between –0.10 and +0.10.

Figure 5A shows cumulative distributions of the subject-specific linear JT/RR slopes. These were steeper in female compared to male subjects (0.175 ± 0.028 vs 0.145 ± 0.023, *p* < 0.0001).

The other panels of Figure 5 show corresponding cumulative distributions of the subject-specific Tpe/RR slopes (Figure 5B corresponds to the Tpe measured in XYZ vector magnitude, the other panels to the Tpe measured in different standard ECG leads). In different leads, these slopes fluctuated around zero. On average, the steepest positive Tpe/RR slopes were found in lead V2 (0.0171 ± 0.0266 and 0.0176 ± 0.0203 in female and male subjects, respectively), the steepest negative Tpe/RR slopes were found in lead V4 (−0.0124 ± 0.0245 and −0.0174 ± 0.0205 in female and male subjects, respectively). Note that the absolute values of even these steepest slopes were approximately only 10% of the JT/RR slopes. In leads III, aVL, V1, V2, and V3, the Tpe/RR slopes were not statistically different between female and male subjects; in all other standard leads, the slope values were higher in female compared to male subjects. In lead V6, the slopes were, on average, positive in female subjects while negative in male subjects (0.0052 ± 0.0128 vs −0.0040 ± 0.0163, *p* = 0.0005).

**FIGURE 6:**
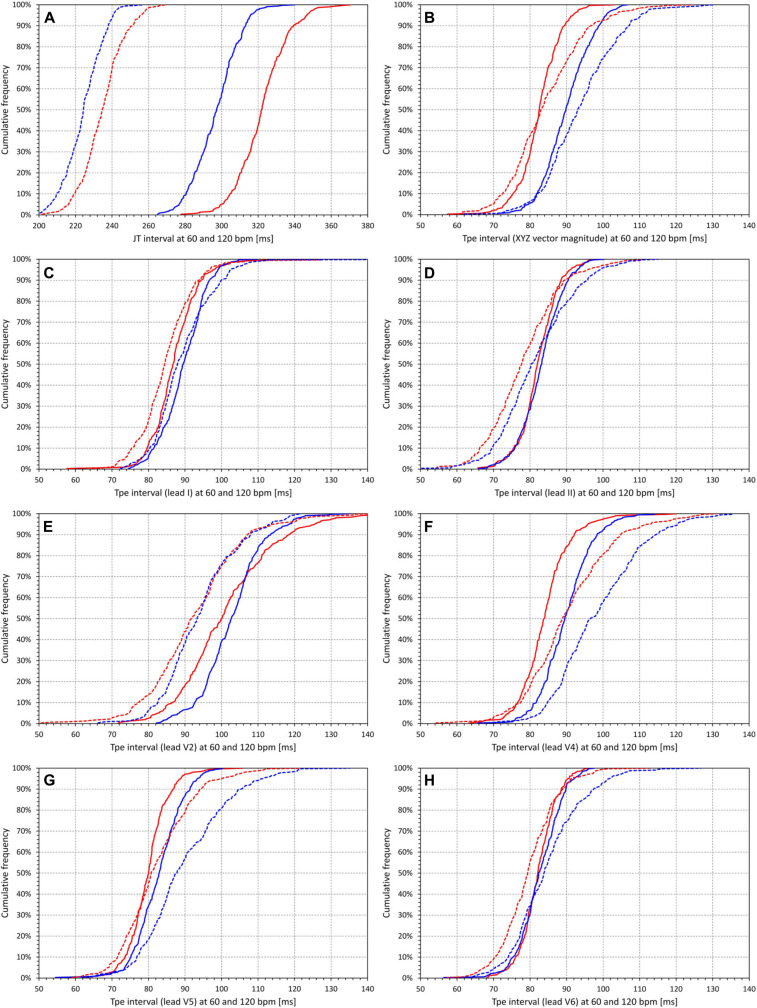
Cumulative distributions of intrasubject regression projections of the **(A)** JT intervals and **(B–H)** Tpe intervals corresponding to heart rate of 60 beats per minute (bpm) (solid lines) and to heart rate of 120 bpm (dashed lines). **(B–H)** correspond to different electrocardiogram (ECG) leads in the same way as the panels of Figure 5. In each panel, the red and blue lines correspond to the distributions in female and male subjects, respectively. Note that while in **(A)**, the horizontal axis ranges between 200 and 380 ms, the horizontal axes in all other panels range between 50 and 140 ms.

The effects of the heart rate influence are summarized in Figure 6. While the heart rate change from 60 to 120 bpm led to JT interval shortening by an average of 87.4 ± 14.1 ms in female and 72.7 ± 11.5 ms in male subjects (*p* < 0.00001, Figure 6A), Tpe interval in the XYZ vector magnitude prolonged, on average, by 1.77 ± 11.19 ms in female subjects and 3.86 ± 9.04 ms in male subjects (*p* = 0.01). In the standard ECG leads, the averaged changes in the Tpe interval again fluctuated around zero. Consistent with the maximum and minimum Tpe/RR slopes, largest averaged shortening of the Tpe interval was found in lead V2 (by 8.55 ± 13.32 and 8.79 ± 10.17 ms in female and male subjects, respectively, *p* = NS for sex comparison), while the largest averaged prolongation was found in lead V4 (by 6.20 ± 12.25 and 8.71 ± 10.25 ms in female and male subjects, respectively, *p* = 0.006 for sex comparison).

Figure 6 also shows that while JT interval was significantly longer in female compared to male subjects (322.3 ± 14.0 vs 297.0 ± 12.6 ms at 60 bpm, and 234.8 ± 11.9 vs 224.3 ± 10.5 ms at 120 bpm, *p* < 0.00001 for both), the opposite was the case for the Tpe interval measured at the XYZ vector magnitude where, on average, the Tpe interval was shorter in female than in male subjects (82.5 ± 5.6 vs 90.0 ± 6.5 ms at 60 bpm, and 84.3 ± 10.3 vs 93.9 ± 10.0 ms at 120 bpm, *p* < 0.00001 for both). In no other lead was the average Tpe interval at either 60 or 120 bpm longer in female than in male subjects, although in some leads (e.g., V2 and V6), the difference between sexes was not statistically significant.

**FIGURE 7:**
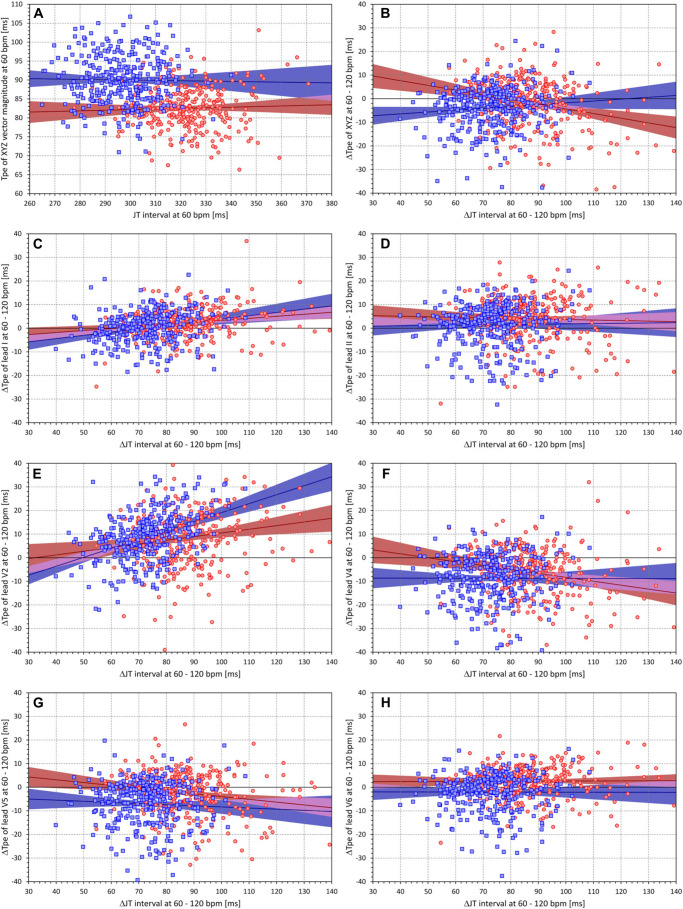
**(A)** Scatter diagram between the subject-specific projections of JT intervals and Tpe intervals (measurement in the XYZ vector magnitude) to the heart rate of 60 bpm. **(B–H)** Scatter diagrams between subject-specific JT interval changes from 60 to 120 bpm and Tpe interval changes from 60 to 120 bpm; the panels correspond to the Tpe measurement in different electrocardiogram (ECG) leads in the same way as the panels of Figure 5. In each panel, the red circles and blue squares correspond to female and male subjects, respectively. The solid red and solid blue lines show the linear regressions between the displayed values in female and male subjects, respectively. The red- and blue-shaded areas are the 95% confidence intervals of the regression lines; the violet areas are the overlaps between the confidence intervals of the sex-specific regressions.

Finally, Figure 7A shows that the intrasubject Tpe interval projections at 60 bpm (measured in the XYZ vector magnitude) were unrelated to the corresponding projections of JT intervals. The same was true for Tpe intervals measured in other ECG leads as well as for the 120-bpm projections (results not shown). The other panels of Figure 7 show that there was no systematic relationship between the 60- and 120-bpm changes of the JT intervals and the corresponding changes in the Tpe intervals measured in different leads. For some leads, the scatter diagrams show positive but weak correlations, while for other leads, weak negative correlations were observed.

### Intrasubject Reproducibility

**FIGURE 8:**
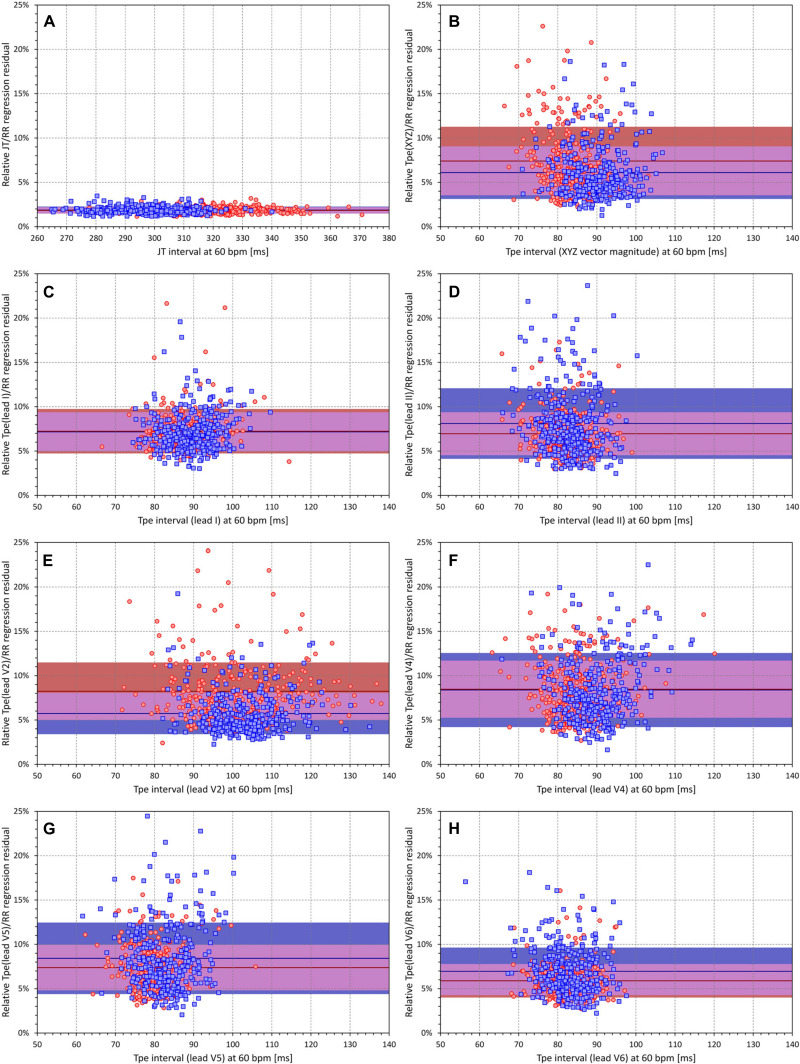
**(A)** Scatter diagram between JT intervals at heart rate of 60 bpm and relative linear JT/RR regression residuals (see the text for explanation). **(B–H)** Similar scatter diagrams between Tpe intervals at heart rate of 60 bpm and relative linear Tpe/RR regression residuals; the panels correspond to the Tpe measurement in different electrocardiogram (ECG) leads in the same way as the panels of Figure 5. In each panel, the red circles and blue squares correspond to female and male subjects, respectively. The solid red and solid blue horizontal lines show the means of the relative regression residuals in female and male subjects, respectively. The red- and blue-shaded areas are the bands of mean ± standard deviation of the relative regression residuals in female and male subjects, respectively. The violet areas are the overlaps between the ±standard deviation bands of both sexes.

Figure 8 shows the comparison of relative Tpe/RR residuals with the relative JT/RR residuals. It is clearly visible that the relative JT/RR residuals were almost one magnitude smaller compared to the Tpe/RR residuals. The smallest relative Tpe/RR residual was seen with the measurements based on lead I (7.24 ± 2.51% and 7.17 ± 2.20% in female and male subjects, respectively, *p* = NS for sex comparison), which was markedly larger (*p* < 0.00001) compared to the relative JT/RR residuals (1.84 ± 0.36% and 1.88 ± 0.39% in female and male subjects, respectively, *p* = NS for sex comparison).

Figure 8 also shows that, while the relative Tpe/RR residuals measured in XYZ vector magnitude were significantly larger in female (7.41 ± 3.86%) than in male subjects (6.09 ± 2.96%, *p* < 0.0001), the same direction of difference existed in some leads (e.g., in lead V2, the residuals were 8.24 ± 3.25% and 5.74 ± 2.33% in female and male subjects, respectively, *p* < 0.0001) but was reversed in other leads (e.g., the residuals in lead V6 were 5.89 ± 1.88% and 6.96 ± 2.68% in female and male subjects, respectively, *p* < 0.0001).

### Reconstruction of Orthogonal Vector Magnitude Tpe From Standard ECG Leads

**FIGURE 9:**
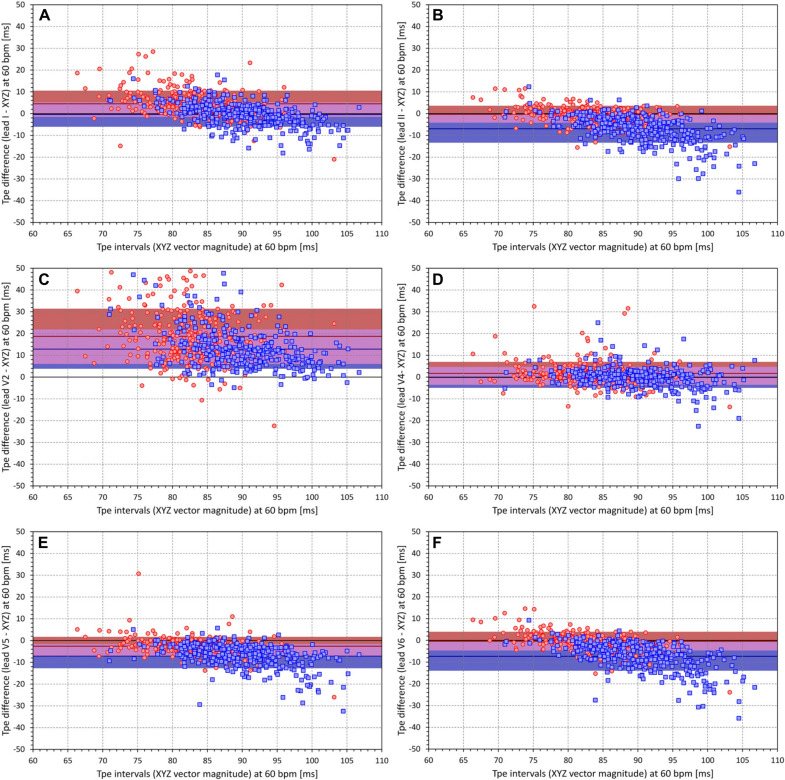
All panels show scatter diagrams between the individual Tpe intervals measured in the XYZ vector magnitude and the corresponding differences between the Tpe intervals measured in different electrocardiogram (ECG) leads and the Tpe intervals measured in XYZ vector magnitude. All the panels show the Tpe values (and their differences) regression projected to the heart rate of 60 bpm. The different panels correspond to the comparison of Tpe measurements made in different ECG leads [**(A)** lead I, **(B)** lead II, **(C)** lead V2, **(D)** lead V4, **(E)** lead V5, **(F)** lead V6]. In each panel, the red circles and blue squares correspond to female and male subjects, respectively. The solid red and solid blue horizontal lines show the means of the Tpe differences in female and male subjects, respectively. The red- and blue shaded areas are the bands of mean ± standard deviation of the Tpe differences in female and male subjects, respectively. The violet areas are the overlaps between the ±standard deviation bands of both sexes.

**FIGURE 10:**
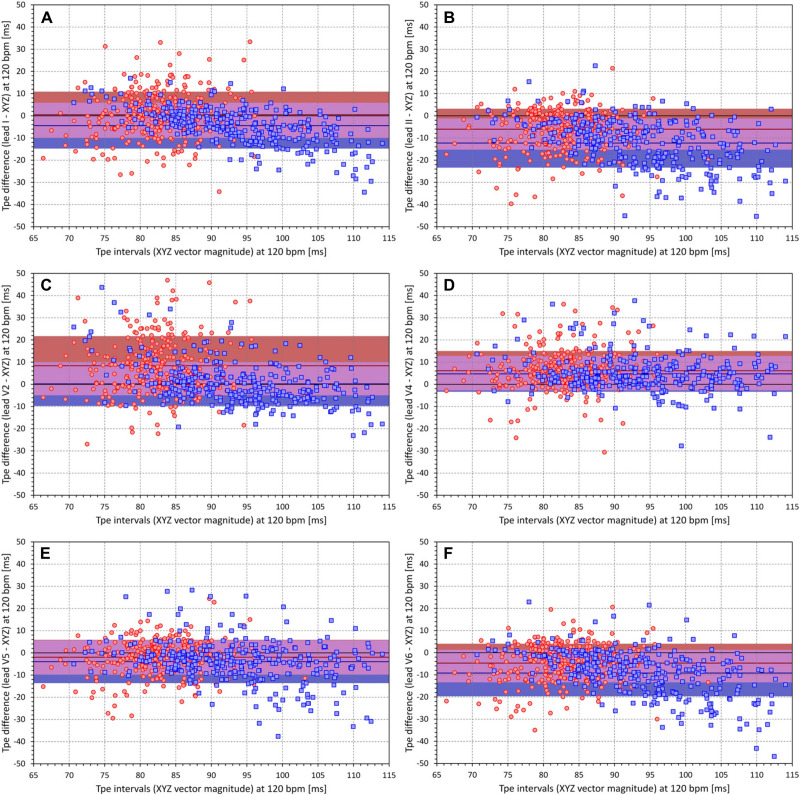
All panels have the same meaning as the panels of Figure 9, but instead of Tpe values individually projected to the heart rate of 60 bpm, values projected to the heart rate of 120 bpm are displayed. Please see the legend of Figure 9 and the labels of the axes for the explanation of panels **(A–F)**.

Figure 9 shows Bland–Altman-like scatter diagram comparing the Tpe interval projections at 60 bpm measured in the XYZ vector magnitude and in standard ECG leads. The figure shows that, while Tpe intervals measured in some leads are closer to the measurement in the XYZ vector magnitude, the relationship to other leads is less clear. The same comparison is shown in Figure 10 that demonstrates the projections at 120 bpm. The spread of corresponding Tpe projections is wider. Hence, there is no standard ECG lead that could be used to obtain a close approximation of the Tpe interval measured in the XYZ vector magnitude.

Of the 796,620 ECG segments investigated in the study, full measurement of the Tpe intervals (i.e., accepted detection of T peaks) in all leads I, II, V2, V3, V4, V5, and V6 was available in 580,430 ECG segments (72.9%). Of these segments, 243,489 were obtained in female subjects (63.2% of all segments in female subjects) and 336,941 were obtained in male subjects (81.9% of all segments in male subjects). Note also that this restriction of the complete measurements was only used in the multivariable regression analyses, while all the previously described results used full datasets of all accepted measurements.

**FIGURE 11:**
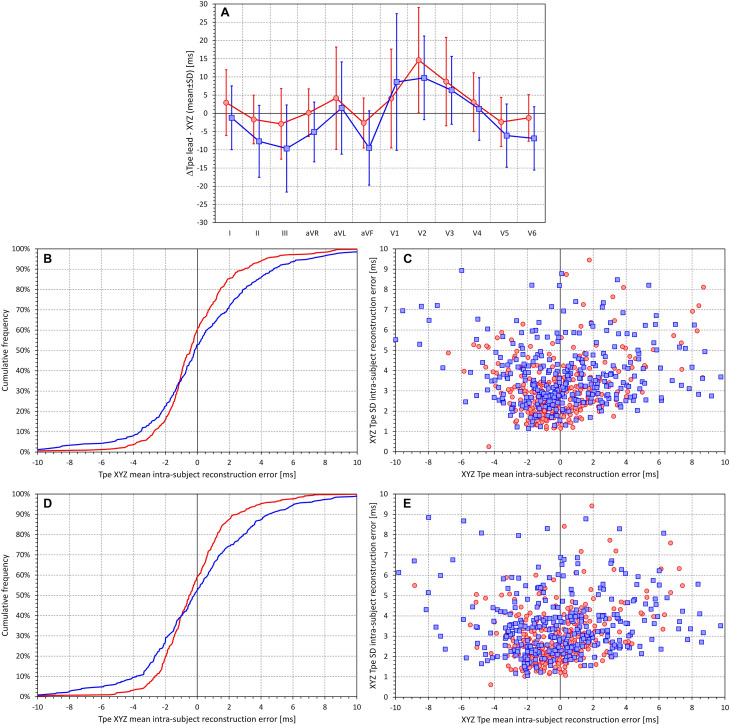
For different electrocardiogram (ECG) leads, **(A)** shows the summaries of the differences between the Tpe measurements in the given lead and the Tpe measurements in the XYZ vector magnitude (analysis based on ECGs with complete measurements pooled together—see the text for details). **(B,D)** Cumulative distributions of the mean intrasubject differences between the Tpe intervals measured in the XYZ vector magnitude and their approximation by the restricted and complete regression models, respectively. **(C,E)** Scatter diagrams between the means and standard deviations of the intrasubject differences between the Tpe intervals measured in the XYZ vector magnitude and their approximation by the restricted and complete regression models, respectively (see the text for details). In **(A)**, the red and blue graphs correspond to the data in female and male subjects, respectively. In **(B,D)**, the red and blue lines correspond to female and male subjects, respectively. In **(C,E)**, the red circles and blue squares correspond to female and male subjects, respectively.

Figure 11A shows the summary of the comparison of the lead measurements of Tpe with the Tpe measurement in XYZ vector magnitude. Although this summary is potentially problematic since it was based on multiples of measurements in the same subjects, trends are seen corresponding to the intrasubject comparisons shown in Figures 9, 10. In some leads (mainly in V2 and V3), the T peak precedes that of the XYZ vector magnitude (making the Tpe interval longer), while in other leads (e.g., II, V5, and V6), the T peak follows that of the XYZ vector magnitude.

Restricted multivariable regression analysis without involving underlying heart rate or an onset constant suggested the following approximation of Tpe interval in XYZ vector magnitude in female subjects:

$$\begin{array}{c} 0.0706{𝔅}_{I}+0.2879{𝔅}_{II}-0.0052{𝔅}_{V2}+0.0313{𝔅}_{V3}\\ +0.2240{𝔅}_{V}4+0.2331{𝔅}_{V5}+0.1625{𝔅}_{V6} \end{array}$$

while the form for male subjects was:

$$\begin{array}{c} 0.1468{𝔅}_{I}+0.1212{𝔅}_{II}+0.0639{𝔅}_{V2}\\ +0.1047{𝔅}_{V3}+0.3339{𝔅}_{V}4+0.2145{𝔅}_{V5}+0.0241{𝔅}_{V6} \end{array}$$

where *𝔅*_*L*_ represents the Tpe interval measured in ECG lead **L** (in ms).

Complete regression analysis proposed the following approximation for female subjects:

$$\begin{array}{c} 17.9237+0.0012𝔔+0.0120{𝔅}_{I}+0.2475{𝔅}_{II}-0.0164{𝔅}_{V2}\\ +0.0328{𝔅}_{V3}+0.2108{𝔅}_{V4}+0.2021{𝔅}_{V5}+0.0894{𝔅}_{V6} \end{array}$$

and for male subjects:

$$\begin{array}{c} 23.7813-0.0002𝔔+0.0924{𝔅}_{I}+0.0855{𝔅}_{II}+0.0005{𝔅}_{V2}\\ +0.1032{𝔅}_{V3}+0.3122{𝔅}_{V4}+0.1855{𝔅}_{V5}-0.0310{𝔅}_{V6} \end{array}$$

where *𝔔* is the RR interval corresponding to the underlying heart rate (in ms).

While in the complete data pooled of all subjects together, these formulae provided close approximations (SD of the differences of 4.089 and 5.606 ms for female and male subjects in the restricted regression and 3.859 and 5.339 ms for female and male subjects in the complete regression), the intrasubject approximations were much less tight. Figures 11B,D show the cumulative distribution of mean approximation differences for the restricted and complete regressions, respectively (i.e., the panels show the distribution of intrasubject errors of these formulae). Corresponding scatter diagrams of the intrasubject means and SDs of the differences between the approximated Tpe intervals measured in the XYZ vector magnitude are shown in Figures 11C,E.

## Discussion

The study leads to four distinct observations and conclusions that appear to be of possible importance for future investigations of the Tpe interval including the assessment of its risk predictive properties.

First, and not surprisingly, the timing of the T peak (and thus the duration of the Tpe intervals) differs in different ECG leads. The spread of the Tpe intervals across ECG leads is related to the spatial width and morphological complexity of the three-dimensional T wave loop. Since T wave loop abnormalities are, together with other T wave morphology indices, known risk predictors (Huang et al., 2009; Hasan et al., 2012; Seegers et al., 2017), it can be expected that in high-risk patients, the lead-to-lead differences in the Tpe interval duration would be larger than in low-risk subjects. Theoretically, it might thus be proposed that a spread of Tpe intervals across leads would approximate T wave loop abnormalities. Nevertheless, since such an approach would have the same technical disadvantages as QT dispersion (Kors and van Herpen, 1998; Malik and Batchvarov, 2000) and since there are more effective and more accurate ways of assessing T wave loop (Acar et al., 1999), this possibility cannot be advocated.

Second, regardless of which ECG lead was used for measurement, we have not found any heart rate dependency of Tpe intervals that would, for practical purposes, require heart rate correction similar to those used for QT or JT intervals. While it has previously been shown that not only the JT intervals but also the J–T peak intervals are substantially and systematically heart rate dependent (Hnatkova et al., 2019b), this is not the case with the Tpe intervals. Using the measurements of any lead, we have found subjects in whom the Tpe correlation with RR of the underlying heart rate was positive and other subjects in whom the correlation was negative (see Figure 5). Proposals have previously been made to correct the Tpe interval using the Bazett formula, arguing that this improves the risk-prediction capabilities of the measurement (Chua et al., 2016). However, such argumentation is misplaced. Resting heart rate is a powerful risk predictor in its own rights (Copie et al., 1996; Böhm et al., 2020) and thus correcting even practically random values by the Bazett formula might lead to significant risk-related population differences (Malik and Camm, 1997).

Third, our results shown in Figure 8 demonstrated substantially poorer intrasubject reproducibility of the Tpe intervals compared to JT intervals (when their durations are related to the underlying heart rate). The intrasubject reproducibility of Tpe measurements is also similarly poorer than that of the QT intervals (results not shown). This is not necessarily surprising since the definition of the end of the T wave is, apart from isoelectric projections of the terminal part of the T wave, independent of the angle projecting the T wave loop into a given ECG lead; the spatial orientation of the T wave loop (and hence the projection of the T wave peak) is influenced by the position of the heart in the thorax, which changes with posture, meal intake, and other circumstances. Since alternative expressions of the distribution of the T wave power were reported to be less variable compared to the T peak identification (Vicente et al., 2017; Hnatkova et al., 2019b), these other expressions are likely worth investigating further. The relatively poor reproducibility of the Tpe interval measurement might also be the reason for inconsistencies in the literature. For instance, while Shenthar et al. (2015) reported Tpe of 200 ± 110 and 100 ± 20 ms in STEMI patients who suffered and did not suffer from ventricular fibrillation, Yu et al. (2018) described Tpe differences of 92.6 ± 11.7 vs 86.8 ± 11.5 ms also among STEMI patients who experienced and did not experience ventricular tachycardia and/or fibrillation.

Finally, our failed attempt of proposing a generally applicable approximation the Tpe intervals in the XYZ vector magnitude based on measurements in standard ECG leads suggests that the interlead differences in the T loop projection are different in different subjects. Considering the individuality of many other repolarization indices, this observation is not surprising. Importantly, the differences in the comparisons of individual lead measurements with those in XYZ vector magnitude (see Figures 9, 10, 11A) show that measurements in different leads should not be mixed. Strategies such as “*If lead V6 was not suitable, leads V5 and V4 were measured.*” (Shenthar et al., 2015) might decrease the stability of measurements, especially if frontal and lateral precordial leads are fused in the same dataset. Our results suggest that, apart from the theoretical considerations that suggest the preference of the XYZ vector magnitude, there are no physiological reasons to prefer one lead over another as long as the same lead is always used. It should also be noted that a number of publications on the Tpe interval stated that V6 is preferable because it best reflects the transmural axis of the left ventricle (Shenthar et al., 2015; Yu et al., 2018). Frequently, a study by El-Sherif et al. (1976) is referenced to support this concept, while surprisingly, this publication by El Sherif et al. does not deal with the topic and provides no credence to the conjecture. Apart from these considerations, the full regression models also showed that RR intervals of underlying heart rate have very little influence on the composite of the Tpe intervals in different leads (note the minimal regression constants of the *𝔔* value contributions).

### Limitations

A number of limitations of our investigation need to be listed. To obtain orthogonal XYZ representation of the ECG signals, we have used previously published conversion matrix optimized for the Mason–Likar electrode positions. Other conversion matrices have also been proposed (Edenbrandt and Pahlm, 1988; Kors et al., 1990) albeit not necessarily suitable for the electrode configuration that was used with the Holter recordings during the source clinical studies. Since the detailed relationship of standard ECG leads to the orthogonal XYZ leads is likely to be subject specific, it might be also possible to use singular value decomposition (Damen and van der Kam, 1982; Acar and Köymen, 1999) and to create an orthogonal lead system specific for each ECG segment. That would, however, potentially complicate the assessment of the relationship to heart rate since different ECGs of the same subject are likely to have different optimal orthogonal projections. When studying the spread of T peak measurements across groups of ECG leads, we have, in addition to standard ECG leads, considered bipolar leads between pairs of electrodes. Other possibilities also exist, e.g., the RA − (V1 + V2)/2 or (V1 + V2)/2 − (V5 + V6)/2, etc. Nevertheless, we believe that considering such possibilities would have been superfluous. While investigating the proportions of the T wave loop, we have not considered T wave amplitude in separate T wave leads. Although the measurements of the end of the T wave were visually validated and manually corrected where appropriate, the identification of T peak positions was based on validated robust algorithm. While the T wave offset was determined in the images of all standard ECG leads superimposed on the same isoelectric axis (Malik, 2004), visual checks of T peak positions would be needed in every lead separately. With the number of ECG segments analyzed in the study, this would have been impossible to achieve. Using the T wave offset common for all ECG leads also eliminated the problems associated with the concept of QT dispersion. We have also not investigated the circadian profile of the Tpe interval duration (Mozos and Filimon, 2013). We have also used a rather simple universally applicable model of Tpe/RR hysteresis (Malik et al., 2016), while other models of hysteresis adaptation were also proposed (Halámek et al., 2010; Gravel et al., 2018). However, it is highly doubtful whether these would have made any difference even if separately applied to the Tpe assessment in different ECG leads. Assessing the hysteresis of RR interval influence properly requires a clear and robust heart rate dependency (Malik et al., 2008b), which we have not found with the Tpe interval. When we repeated the same data analyses using only ECG segments preceded by stable heart rate (results not shown), the same absence of any systematic heart rate dependency was found. The fact that, in individual leads, the Tpe measurements were more frequently accepted in male compared to female individuals reflects the slightly higher noise pollution of Holter recordings in female individuals, which, compared to male individuals, leads also to marginally lower intrasubject QTc variability (Malik et al., 2013).

## Conclusion

Despite these limitations, the study leads to the following conclusions. First, even in normal healthy recordings, the Tpe intervals differ across ECG leads, and the spread of their durations is related to the spatial width and morphological complexity of the three-dimensional T wave loop. Second, irrespective of the measured ECG lead, the duration of Tpe interval is not systematically heart rate dependent; no heart rate correction should be used in the clinical Tpe investigations. Third, compared to other repolarization-related intervals, the Tpe measurement suffers from poorer intrasubject reproducibility. Finally, the relationship between Tpe intervals measured in different ECG leads is different in different subjects; studies of the Tpe intervals should therefore avoid combining measurements in different ECG leads.

## Data Availability Statement

The raw data supporting the conclusions of this article will be made available by the authors, without undue reservation but pending the approval by the sponsors of the source clinical studies.

## Ethics Statement

The studies involving human participants were reviewed and approved by the Focus in Neuss; Parexel in Baltimore; Bloemfontein, and Glendale; PPD in Austin; and Spaulding in Milwaukee. The patients/participants provided their written informed consent to participate in the source studies.

## Author Contributions

IA, KH, and MM: study design and initial manuscript draft. KH and MM: software development and, statistics and figures. GS, IA, KMH, MŠ, OT, PB, PS, and TN: ECG interpretation and measurement. GS, TN, OT, and PS: supervision of the measurements. GS, MM, and TN: quality control of the measurements. All authors contributed to the article and approved the submitted version.

## Conflict of Interest

The authors declare that the research was conducted in the absence of any commercial or financial relationships that could be construed as a potential conflict of interest.
